# Characterization and phylogenetic analysis of the chloroplast genome of *galium spurium*

**DOI:** 10.1080/23802359.2023.2172971

**Published:** 2023-03-29

**Authors:** Hanbin Yin, Kerui Huang, Peng Xie, Ping Mo, Ningyun Zhang, Yun Wang

**Affiliations:** Hunan Provincial Key Laboratory for Molecular Immunity Technology of Aquatic Animal Diseases, College of Life and Environmental Sciences, Hunan University of Arts and Science, Changde, China

**Keywords:** *Galium spurium*, chloroplast genome, phylogenetic analysis

## Abstract

*Galium spurium* is a farmland weed, with strong stress resistance. However, its chloroplast genome has never been reported. In this study, the complete sequence of the chloroplast genome of *G. spurium* was characterized, which is a circular molecule, 153,481 bp in length, and with a large single copy region of 84,334 bp, a small single copy region of 17,057 bp, and a pair of inverted repeat regions of 26,045 bp. The whole genome contained 127 genes, including 82 protein-coding genes, 37 transfer RNA genes, and eight ribosomal RNA genes. Phylogenetic analysis shows that it relates closely to *G. aparine*. This study provides a basis for the further phylogenic study of *Galium.*

## Introduction

**Figure 1.**
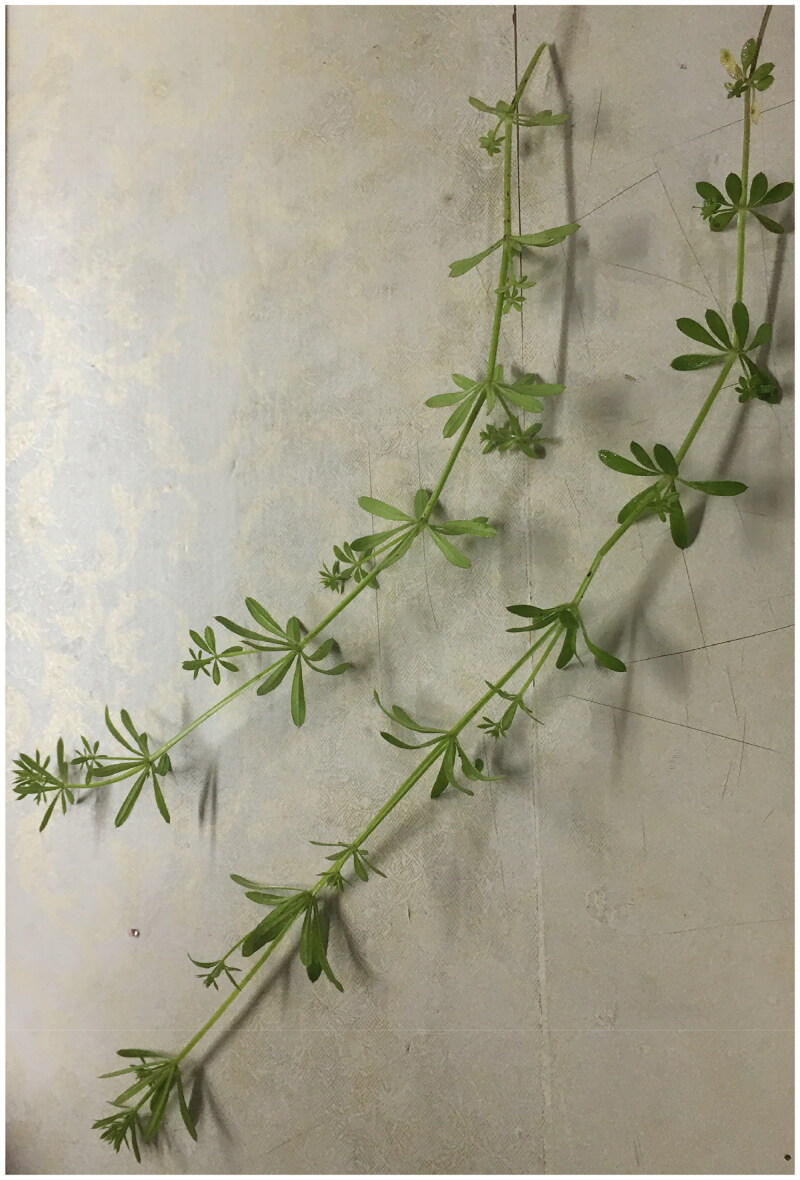
Picture of the collected sample of *Galium spurium*. Note: The picture is self-taken, the sample is collected from the Fourth Canteen of the Hunan University of Arts and Sciences, Changde, Hunan Province, China (29°03'08.68″N, 111°39'47.46″E, 33m)

*Galium spurium* Linnaeus 1753 is a trailing or climbing annual herb of the Rubiaceae family, which grows in fields, riversides, farmland, and hillsides near sea level to 4600 m in almost all parts of China (Figure 1). It is also scattered throughout Canada, the Mediterranean, and the European continent. *G. spurium* stems are four-angled with retrorsely aculeate and glabrescent to pilose at nodes. Leaves occur at the middle stem region in whorls of 6–8, subsessile; blade drying papery, narrowly oblanceolate to narrowly oblong-oblanceolate (Wu et al. 2011). The vitality of *G. spurium* is highly tenacious. It has high seed-setting and fast growth rates, which can capture large photosynthetic areas against crops. Furthermore, *G. spurium* can rejuvenate, that is, it has two life cycles; only the early autumn frost causes the plant to die. In this context, *G. spurium* has developed from a minor weed to the main weed. *G. spurium* significantly threatens rape cultivation, and the increase in its invasion correlates positively with increased rapeseed planting areas. Recently, with excessive herbicide usage, some *G. spurium* has developed resistance, and the seeds of the species have been classified as harmful according to Seeds Regulations (Beckie et al. 2020). However, despite the negative effects of *G. spurium*, it is medicinally valuable. The aerial part of the plant is used to treat bones. It clears away heat, detoxifies, aids diuresis, and lowers blood pressure. Moreover, it is rich in chemical components, including alkaloids, anthraquinones, flavonoids, iridoids, naphthalene derivatives, triterpenoid saponins, etc (Ahn and Kim 2012). Although there are several reports about the weed in certain fields, little is known about its phylogenetic research, and its chloroplast genome remains unclarified. In this study, the characterization (sequencing, assembly, and annotation) and phylogenetic analysis of the chloroplasts of *G. spurium* were conducted, which help to explore the phylogenetic status of *G. spurium* and provide information for the mining of the potential value of this species.

## Materials

Leaf samples of *G. spurium* were collected from the lawn of the Fourth Canteen of the Hunan University of Arts and Sciences, Changde, Hunan Province, China (29°03'08.68"N, 111°39'47.46"E, 33 m). The voucher specimens were well preserved, and are accessible at the specimen storage room of the College of Life and Environmental Sciences, Hunan University of Arts and Sciences (contact person: Kerui Huang, huangkerui008@163.com, voucher number ZYY012).

## Methods

The total genomic DNA extraction and sequencing process followed the method of Mo *et al.* (Mo et al. 2022). A total of 72,260,228 reads were excluded after filtering out the low-quality reads using fastp (Chen et al. 2018). Then, using GetOrganelle v1.7.5 (Jin et al. 2020), the *G. spurium* chloroplast genome was assembled *de novo*. Finally, CPGAVAS2 (Shi et al. 2019) was used to annotate the chloroplast genome and CPGView (http://www.1kmpg.cn/cpgview/) was used to draw the genome map. The phylogenetic analysis was performed as follows: 33 chloroplast genomes with the closest relationship to *G. spurium* were obtained from GenBank, 58 common protein genomes in all genomes were screened, and then each gene was aligned individually using MAFFT v7.313 (Rozewicki et al. 2019). Then, each gene was processed using Gblocks 0.9b and linked end-to-end into a supergene for each species using all genes (Guo et al. 2022). Maximum-likelihood phylogenies were inferred using IQ-TREE v1.6.8 (Nguyen et al. 2015) under the GTR + F + I + G4 model for 5000 ultrafast bootstraps.

## Results

**Figure 2.**
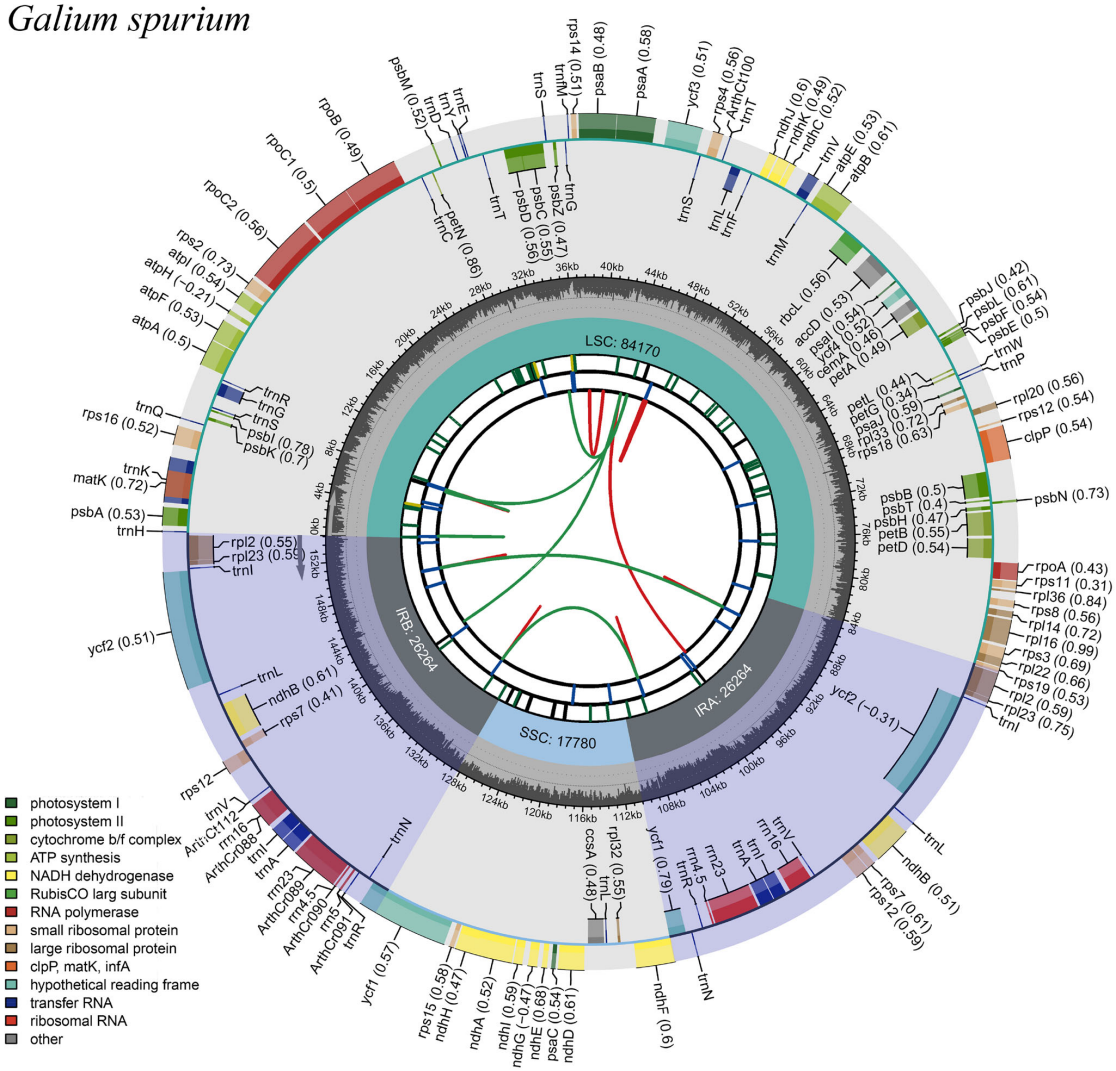
Gene map of the *Galium spurium chloroplast genome.*

The chloroplast genome structure of *G. spurium* is a circular molecule, 153,481 bp in length, with a large single copy (LSC) region of 84,334 bp, a small single copy (SSC) region of 17,057 bp, and a pair of inverted repeat (IR) regions of 26,045 bp (Figure 2). The total G + C content of the chloroplast genome was 37.17%, whereas the GC contents of LSC, SSC, and IR regions were 34.77%, 31.21%, and 43.03%, respectively. The genome contained 127 genes, including 82 protein-coding genes, 37 transfer RNA genes, and eight ribosomal RNA genes, of which 18 were duplicated in the IR regions.

**Figure 3.**
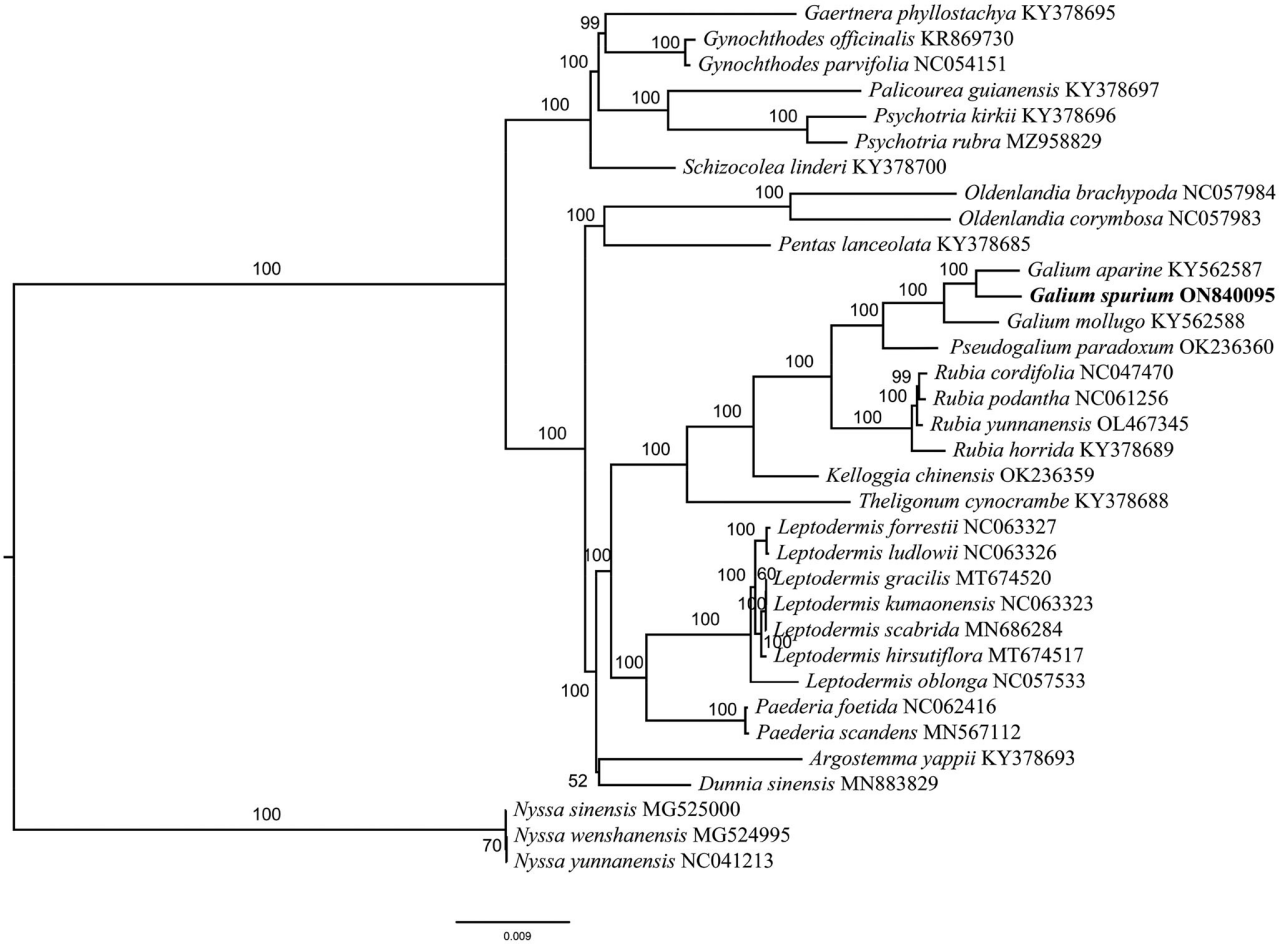
A maximum-likelihood tree of *Galium spurium* and 33 related species was reconstructed by using the IQ-Tree based on 58 protein-coding genes shared by all genomes. Bootstrap values are shown next to the nodes.The following sequences were used: Gaertnera phyllostachya KY378695 (Wikström et al. 2020), Gynochthodes officinalis KR869730 (Zhang et al. 2016), Gynochthodes parvifolia NC054151 (Cai et al. 2020), Palicourea guianensis KY378697 (Wikström et al. 2020), Psychotria kirkii KY378696 (Wikström et al. 2020), Psychotria rubra MZ958829 (Geng et al. 2022), Schizocolea linderi KY378700 (Wikström et al. 2020), Oldenlandia brachypoda NC057984 (Varani et al. 2022), Oldenlandia corymbosa NC057983 (Varani et al. 2022), Pentas lanceolata KY378685 (Wikström et al. 2020), Galium aparine KY562587 (Dann et al. 2017), Galium mollugo KY562588 (Dann et al. 2017), Pseudogalium paradoxum OK236360 (Varani et al. 2022), Rubia cordifolia NC047470 (Zhao et al. 2020), Rubia yunnanensis OL467345 (Wang et al. 2022), Rubia horrida KY378689 (Wikström et al. 2020), Kelloggia chinensis OK236359 (Yang et al. 2022), Theligonum cynocrambe KY378688 (Wikström et al. 2020), Leptodermis forrestii NC063327 (Zhang et al. 2021), Leptodermis ludlowii NC063326 (Zhang et al. 2021), Leptodermis gracilis MT674520 (Zhang et al. 2021), Leptodermis kumaonensis NC063323 (Zhang et al. 2021), Leptodermis scabrida MN686284 (Zhang et al. 2020), Leptodermis hirsutiflora MT674517 (Zhang et al. 2021), Leptodermis oblonga NC057533 (Guo et al. 2021), Paederia scandens MN567112 (Li et al. 2019), Argostemma yappii KY378693 (Wikström et al. 2020), Dunnia sinensis MN883829 (Zhang et al. 2020), Nyssa sinensis MG525000 (Fu et al. 2017), Nyssa wenshanensis MG524995 (Fu et al. 2017), Nyssa yunnanensis NC041213 (Yang et al. 2019).

The phylogenetic analysis showed that *G. spurium* is closely related to *G. aparine, with 100% support (*Figure 3), which is generally consistent with previous studies (Jeong et al. 2016; Ehrendorfer et al. 2018).

## Discussion and conclusion

This is the first study to report the characterization of the chloroplast genome of G. spurium, and the phylogenetic relationship between *G. spurium* and other species of *Galium* was generally consistent with previous studies (Jeong et al. 2016; Ehrendorfer et al. 2018). However, as only a few chloroplast genomes of *Galium* have been reported, and as data in the public database are insufficient, the phylogeny of *Galium* requires further study. This study provides useful information to studying the phylogenetic evolution of *G. spurium.*

## Data Availability

The complete chloroplast genome sequence of *Galium spurium* has been deposited in the GenBank database under the accession number ON840095 (https://www.ncbi.nlm.nih.gov/nuccore/ON840095.1). The associated BioProject, SRA, and Bio-Sample numbers are PRJNA872853, SRR21185890, and SAMN30471510, respectively.
